# The role of histone methylase and demethylase in antitumor immunity: A new direction for immunotherapy

**DOI:** 10.3389/fimmu.2022.1099892

**Published:** 2023-01-11

**Authors:** Yuanling Zhang, Junhao Chen, Hang Liu, Rui Mi, Rui Huang, Xian Li, Fei Fan, Xueqing Xie, Jie Ding

**Affiliations:** ^1^ School of Medicine, Guizhou University, Guiyang, China; ^2^ Department of Gastrointestinal Surgery, Guizhou Provincial People’s Hospital, Guiyang, China; ^3^ Graduate School of Zunyi Medical University, Zunyi, China; ^4^ Department of Medical Cosmetology, Guizhou Provincial People’s Hospital, Guiyang, China; ^5^ Department of General Surgery, Zhijin County People’s Hospital, Bijie, China; ^6^ Orthopedics Department, Dongguan Songshan Lake Tungwah Hospital, DongGuan, China; ^7^ Department of Thyroid and Breast Surgery, Affiliated Hospital of Panzhihua University, Panzhihua, China

**Keywords:** histone methylation, epigenetic modification, tumor, antitumor immunity, immunotherapy

## Abstract

Epigenetic modifications may alter the proliferation and differentiation of normal cells, leading to malignant transformation. They can also affect normal stimulation, activation, and abnormal function of immune cells in the tissue microenvironment. Histone methylation, coordinated by histone methylase and histone demethylase to stabilize transcription levels in the promoter area, is one of the most common types of epigenetic alteration, which gained increasing interest. It can modify gene transcription through chromatin structure and affect cell fate, at the transcriptome or protein level. According to recent research, histone methylation modification can regulate tumor and immune cells affecting anti-tumor immune response. Consequently, it is critical to have a thorough grasp of the role of methylation function in cancer treatment. In this review, we discussed recent data on the mechanisms of histone methylation on factors associated with immune resistance of tumor cells and regulation of immune cell function.

## Introduction

Over the past decade, immunotherapy, such as immune checkpoint and CAR T cell therapy, has become a promising strategy for treating cancer (1, 2). Cancer treatment is achieved by increasing the number and effectiveness of immune cells, which can recognize tumor cells, collaborating with tumor surface suppressors and soluble factors in the tumor microenvironment to prevent the tumor invasion and metastasis, thus maintaining the immune microenvironment homeostasis of the body, and improving immune response (3–5). However, due to the tumor heterogeneity and primary or acquired treatment resistance, only 10% to 30% of patients can benefit from immunotherapy (6–8). Therefore, identifying the source of low immune reactivity, effectively regulating immune cell and tumor cell therapeutic targets, and improving immunogenicity are of utmost importance.

The oncogenic transformation caused by the accumulation of related oncogene and tumor suppressor gene mutations accompanied by alteration of histone methylation modification has been observed in various human cancers, further emphasizing the importance of histone methylation modification in medical oncology research (9, 10). Many studies have suggested that aberrant methylation of histones can reduce the expression of tumor-associated antigens, hinder antigen presentation, and affect the exercise of anti-tumor immunity by anti-tumor effector T cells, specialized antigen-presenting cells (APCs), and other cells (11, 12). Moreover, it can alter the number and differentiation process of non-specialized APC infiltration, such as myeloid-derived suppressor cells (MDSCs), regulatory T cells (Tregs), and tumor-associated macrophages (TAMs), assisting tumor cell immune escape (13). Given the impact of histone methylation modification on the immune system and tumor cells, it is worth exploring whether targeting these enzymes may alter the tumor immune microenvironment and improve the efficacy of immunotherapy. Our findings showed that enzymes involved in histone methylation regulate tumor immunity, providing innovative strategies for formulating more perfect immunotherapy strategies. In this review, we discussed the effect and mechanism of aberrant histone methylation in the tumor immune microenvironment on immune cells and tumor cells.

## Classification and biological functions of histone methyltransferases (HMTs)

The amino terminus of histones can be modified to create a class of “histone codes” that increase the amount of information in the genetic code of genes, resulting in different cell fate and pathological development in the same cases (14). Lysine and arginine residues of certain histones are catalyzed by a family of conserved proteins known as the histone methyltransferases (HMTs), consisting of two species based on their structure and modification sites, i.e., histone lysine methyltransferase (KMT) and protein arginine methyltransferase (PRMT), both of which use N-terminal residues as modification sites, such as H3K4, H3K9, H3K27, H3K36, H3K79, and H4K20 (15). Most KMT contain a conserved catalytic domain, called the SET domain. Accordingly, the KMT family can be divided into SET domain-containing enzymes, including EZH2, G9a, SETD2, SUV39H1, and SET domain-free DOT1-like proteins (16). PRMT is a group of enzymes that use S-adenosine methionine (SAM) as a methyl donor. The PRMT family has nine members (PRMT1-9) that generate a single methyl group, which is added to the target protein to create a monomethylarginine (MMA) tag (17). Based on the catalyzed methylation reaction type, the PRMT family is divided into three isoforms, a class of highly conserved genetic products (18).

HMTs have a major role in the epigenetic regulation of gene expression, especially in the regulation of genes related to tumor invasion and metastasis. HMTs catalyze the lysine and arginine residues of particular histones, which are involved in a variety of biological activities, including packaging of chromosome structures, affecting transcription factor recruitment and binding, initiation and extension factors and target DNA binding, RNA processing, editing, and other processes. They also regulate genome mutations, ultimately leading to cancer (10). These methyltransferases have been demonstrated to have an important role in tumor maturation, carcinogenesis, and maintenance of stem cell components. HMTs act in a closely controlled manner to direct the necessary cellular processes under normal cell physiological settings. However, these enzymes may dysregulate and modify the epigenetic landscape and proteome to drive cell growth and survival in malignant circumstances (18, 19).

### Histone lysine methyltransferase (KMT) and tumor immunity

**Figure 1:**
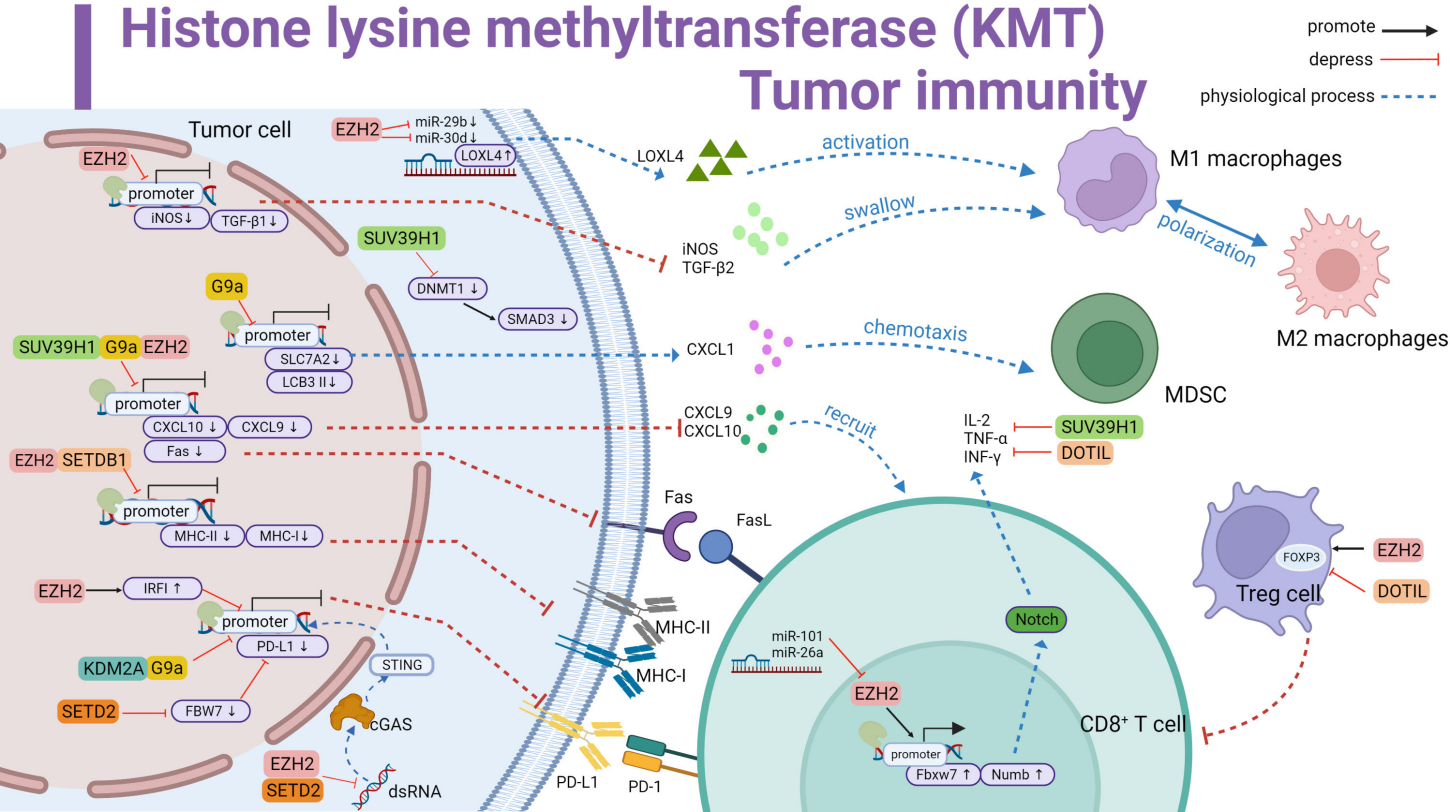
Histone lysine methyltransferase (KMT) involved in tumor immune summary. Promotes LOXL4 upregulation by antagonizing miR-29b and miR-30d to activate macrophage polarization; downregulates iNOS and TGF-β1 and inhibits macrophage phagocytosis. Downregulates iNOS and TGF-β1, inhibits macrophage phagocytosis. G9a inhibits SLC7A2, upregulates CXCL1 and thus recruits MDSC. G9a inhibits LCB 3II transcription and promotes immune escape. SUV39H1, G9a and EZH2 inhibit CXCL10 and CXCL9 transcription and reduce T cell recruitment. Meanwhile inhibit Fas transcription and curb Fas-FasL signaling pathway activation. EZH2 and SETDB1 repress MHC-II and MHC-I to affect antigen recognition. KDM2A and G9a directly repress the initiation of PD-L1 transcription. EZH2 and SETD2 inhibit the dsRNA-cGAS-STING pathway in the cytoplasm affecting PD-L1 transcription. In addition, EZH2 upregulates IRF1 to inhibit PD-L1 transcription. SETD2 downregulates FBW7 to inhibit PD-L1 expression. SUV39H1 inhibits SMAD3 in the cytoplasm and forms immunosuppression. EZH2 promotes FOXP3 transcription and Treg cell suppressor function. In contrast, DOTIL is the opposite. In T cells, EZH2 upregulates IL-2,TNF-αand INF-γ by promoting Fbxw7 and Numb activation of the Notch pathway, a process inhibited by miR-101 and miR-26a antagonism. SUV39H1 and DOTIL suppress the expression of immune factors. Black line represents promotion, red line represents inhibition, and dashed line represents physiological function.

KMT abnormalities in the complex tumor microenvironment cause expression mutations of key immune regulators in tumor cells and effector genes in immune cells, which may lead to antigen presentation suppression, loss of immune tolerance, blocked anti-tumor immunity, and negative effects on immunotherapy. In the following paragraphs, we discuss the regulatory mechanisms of numerous popular histone lysine methylases in tumors and their effect on immune cells, further emphasizing the crucial necessity of inhibiting histone lysine methylases for immunotherapy (**Table 1**) (**Figure 1**).

**Table 1:** Related functions of lysine methylase and tumor immunity.

Protein	Tumor type	Regulate cell	Target	Mechanistic	References
EZH2	Diffuse large B-cell lymphoma	Tumor cell	MHC-I/MHC-II	Inhibition of both MHC-I and MHC-II expression	(20)
	Pan cancer	Tumor cell	MHC-I	Down-regulate MHC-I expression	(21)
	Prostate cancer	Tumor cell	STING	Blocking the activation of RNA-STing-ISG stress response	(22)
	Hepatocellular carcinoma	Tumor cell	IRF1	Suppress PD-L1 expression by upregulating the promoter H3K27me3 levels of CD274 and IRF1	(23)
	Breast cancer	Macrophage	miR-29b/miR-30d	Promoting LOXL4 expression through repressing the expression of miR-29b and miR-30d to regulating macrophage activation	(24)
	Glioblastoma multiforme	Macrophage	iNOS/TGFβ2	Inhibition of EZH2 activates iNOS and increases TGFβ2 levels to enhance phagocytic activity and survival of microglia	(25)
	Ovarian cancer/Colon cancer	CD8^+^ T cell	CXCL9,CXCL10	Affects T cell migration *via* controlling the expression of CXCL9 and CXCL10	(26, 27)
	Pan cancer	T cell	ARID1A	Combines with ARID1A to restore CXCL9 and CXCL10 expression and promote T cell infiltration	(28)
	Colorectal	Treg cell	N/A	Control H3K27me3 levels to block antitumor T cell responses	(29)
	cancer Ovarian cancer	T cell	Numb,Fbxw7	Activate Notch pathway and stimulate T cell polyfunctional cytokine expression	(30)
	N/A	CAR T cell	N/A	Remodeling the epigenome associated with CAR T cell exhaustion	(31)
G9a	Melanoma/Colon cancer	Tumor cell	N/A	Inhibit the IFN-induced expression of the CXCL9 and CXCL10	(32, 33)
	Oophoroma	Tumor cell	N/A	Involved in inhibiting the expression of multiple chemokines	(34)
	Melanoma	Tumor cell	LC3B II	Increase H3K9 enrichment in the LC3B II promoter region and decrease immune blocker reactivity	(35)
	Colon Carcinoma	Tumor cell	Fas	Restrict the transcriptional initiation of Fas and limit the release signal of Fas-FasL	(36)
	Hepatocellular carcinoma	Tumor cell	SLC7A2	Downregulation of SLC7A2 induces MDSO chemotaxis *via* CXCL1	(37)
SETDB1	Melanoma/Lung cancer	Tumor cell	TE	Derepresses TEs to generate MHC-I peptides and triggers T-cell responses	(38)
	Pan cancer	Tumor cell	PD-L1	Inhibit PD-L1 expression and reduce T cell infiltration	(39, 40)
	Pan cancer	Tumor cell	TE	Disruption of TEs promptes cells to maintain cancerous state	(41, 42)
SUV39H1	Cervical carcinoma	Tumor cell	DNMT1	H3K9me2 interacts with the DNMT1 promoter region to affect downstream SMAD3 expression	(43)
	N/A	T cell	N/A	Expression of the silent memory genes	(44)
	N/A	T cell	SMAD3	Interacts with Smad3 and enhances the IL-2 promoter repressor activity	(45)
SETD2	Pan-cancer	N/A	N/A	Participate in the efficacy of immunotherapy	(46)
	Lung adenocarcinoma	N/A	N/A	Enrichment of the mutations involved in PD-L1	(47)
	Renal cell carcinoma	Tumor cell	FBW7	Increase PD-L1 expression by targeting the FBW7/NFAT1 axis	(48)
KMT2A	Pancreatic cancer	Tumor cell	CD274	Directly binds to the CD274 promoter to catalyze H3K4me3 to activate PD-L1 transcription in tumor cells	(49)
	Hepatocellular carcinoma/Nonsmall cell lung cancer	N/A	N/A	Mutations areassociated with PD-L1	(50, 51)
	Pan-cancer	N/A	N/A	Participate in immune regulation	(52–57)
DOT1L	N/A	T cell	TCR	Controll CD8 T cell differentiation by ensuring normal T cell receptor density and signaling	(58, 59)
	Colorectal cancer	Treg cell	N/A	Altering the T cell subsets	(60)

#### EZH2

The Zeste homology 2 (EZH2) is responsible for modifying the lysine methylation of histone 3 (H3K27me3) to silence the gene (61). Previous studies have shown that EZH2 participates in malignant biological phenotypes such as the cell cycle, proliferation, invasion and metastasis actin, which is an important target for solid tumors and hematological tumors (62, 63). Moreover, several potential molecular mechanisms have revealed that EZH2 enrichment shapes the immunosuppressive tumor microenvironment. In tumor cells, EZH2 mutations down-regulate the expression of tumor antigens, thereby evading specific immune recognition by T cells. Major histocompatibility complex-I (MHC-I) acts as a potent marker for T cells to monitor tumors sensitively, and EZH2 suppresses its normal expression. Treatment with EPZ-6438 or EPZ-011989, EZH2 inhibitor, significantly depleted H3K27me3 and increased the expression of surface MHC-I protein (20, 21). In addition, studies have shown that the overexpression of EZH2 can inhibit programmed cell death protein 1 (PD-L1) in prostate cancer and hepatocellular carcinoma by enhancing the H3K27me3 level of the interferon regulatory factor 1(IRF1) transcription factor (22, 23). The use of EZH2 inhibitors (EPZ) activates the STING stress response to promote INF-γ-induced PD-L1 expression. Furthermore, EZH2 inhibitor combined with PD-1 treatment did not produce resistance or toxicity and had significant therapeutic effects (22).

EZH2 can also drive tumor cells to release certain mediators to affect the transport and activity of immune cells. LOXL4 is an important chemical inducer of macrophages. It was reported that EZH2 regulates macrophage activation through the miR-29b/miR-30d-LOXL4 axis and enhances tumor-associated macrophage (TAM) infiltration in breast cancer (24). In glioblastoma multiforme (GBM), iNOS and TGF-β2 can impaire engulfing and viability of macrophages (25). The number of infiltrating cells and the lethality of T cells represent the improved anticancer immunity of the body. Genome-wide studies showed that EZH2 levels are negatively correlated with CD8^+^ T cells, mainly inhibiting the production of tumor TH1-type chemokines CXCL9 and CXCL10 and thus reducing the recruitment of T cells (22, 26, 27), while the binding of carboxyl structure of ARID1A to EZH2 can reverse this step (28). Animal experiments have shown that the synergistic treatment of ovarian cancer with GSK126 (EZH2 inhibitor) and DNMT inhibitor improves the therapeutic efficacy of anti-PD-L1 therapy and overt T-cell therapy (27). In additional, the use of CPI-1205 (EZH2 inhibitor) in a mouse colorectal cancer tumor (MC38) model had a synergistic effect on the immunotherapeutic modality (29). Meanwhile, the activity of EZH2 in Treg cells maintains the stability of FOXP3 protein, increases the number of tumor-infiltrating FOXP3^+^ Tregs, alters the homeostatic balance with tumor effector T cells in the microenvironment and impairs the anti-tumor immune response (29). In contrast, EZH2 in CD8^+^ T cell can activate the Notch pathway, promote the release of cytokines in T cells, and maintain its good antineoplastic activity (30). Moreover, EZH2 is also involved in genome remodeling related to T-cell failure and promotes functional recovery (31). However, the tumor microenvironment can limit the conversion of oxidative phosphorylation to aerobic glycolysis by maintaining high expression of microRNA101 and microRNA26a, and limit the expression of EZH2 in T cells by controlling glucose metabolism. This hinders the normal expression of multifunctional cytokines (30). In overview, EZH2 has an important regulatory role on immune microenvironment components. Several clinical trials are currently recruiting to test the CPI-1205 or tazemetostat (an EZH2-targeted agent) in combination with Pembrolizumab in solid tumors (NCT03854474 and NCT03337698).

##### G9a

G9a (Euchromatic histone-lysine N-methyltransferase 2, EHMT2) is frequently upregulated in different types of cancer (64). G9a overexpression enhances H3K9me2 deposition, silencing and inhibiting tumor suppressor genes, and promoting tumor proliferation and migration through the Wnt pathway and epithelial-to-mesenchymal transformation (EMT), which can be a useful target for anticancer therapy (65). Notably, the special effects of G9a and the tumor microenvironment (TME) may explain the poor immunogenicity in specific cancers. For example, G9a is inversely associated with CD8^+^ T cell infiltration in melanoma and colon cancer. Moreover, it can inhibit the activated of Th1 cytokines/chemokines (32, 33). Further investigation revealed that Ga9 induces chromatin variability in chemokine-related genes, involved in homing of intratumoral effector lymphocytes and natural killer cells (34). In clinical cases, immunohistochemistry showed high intensity of G9a staining in 12 melanoma patients who did not respond to anti-PD-1 or anti-CTLA-4 treatment. Mouse melanoma resistance models treated with UNC0642 (a G9a inhibitor) in combination with anti-PD-1 therapy significantly reduced H3K9 levels in the LC3B II promoter region activating cellular autophagic responses and increasing PD-L1 levels, enhancing the blockade response to PD-1 immune checkpoint inhibitors (35).

G9a can also influence the methylation levels of multiple activated molecules of immune-related pathways. A previous study showed that G9a enhances H3K9me3 enrichment in the Fas promoter, restricts Fas-fasL release signals, and inhibits the tumor immune surveillance of host T cells (36). Moreover, in hepatocellular carcinoma (HCC), G9a silences SLC7A2 expression to induce CXCL1, promoting the recruitment of bone marrow-derived suppressor cells (MDSC) to the microenvironment (37). Given the above regulatory mechanisms, inhibition of G9a can remodel active tumor antigens and substantially modulate the tumor immune microenvironment. The combination of G9a inhibitors and immunotherapy strategies may be able to convert some “cold” immune tumors into “hot” tumors to achieve good immunotherapeutic results.

#### SETDB1

The Forked histone lysine methyltransferase 1 (SETDB1) containing the SET domain is responsible for the di-and trimethylation of the H3K9 residues. It is abnormally amplified and overexpressed in tumors (66). Yet, the underlying mechanisms of SETD2 gene mutations or loss of function leading to the corresponding dysfunction of tumor tissue proteins remain largely unexplored. Animal experiments showed that accumulation of SETDB1 mutations downregulates MHC-I-associated antigen presentation, thus preventing CD8^+^ T from correctly recognizing tumor cells and affecting sensitivity to PD-1/CTLA-4 treatment (38). On the other hand, SETDB1 in tumor cells forms a complex with TRIM28 or acts together with KDM5B that interferes with PD-L1 expression by blocking double-stranded RNA (dsRNA) production through the endogenous retroviral (ERV) pathway (39, 40). The loss of the SETDB1 gene also triggers type I interferon-induced PD-L1 expression through the cyclic GMP–AMP synthase (cGAS)–stimulator of interferon genes (STING) pathway and enhances anti-PD-L1 immune checkpoint blockade for antitumor effects (39–42). cGAS-STING pathway, an important pathway regulating host innate immunity, has been successively validated in various tumor models where SETD2 is an important epigenetic regulator. Thus, SETD2 is an attractive target for promoting immunotherapeutic responses.

#### SUV39H1

The variant suppressor 39 homolog 1 (SUV39H1), also known as KMT1A, is responsible for the introduction of the dimethylation and trimethylation of histone 3 lysine 9 (H3K9me3) (67). It mainly disrupts some important gene regulatory elements in tumor cells and reduces the sensitivity to immune response. In cervical cancer, SMAD3 is a key mediator of activation of multiple immune signaling pathways. SUV39H1 negatively regulates DNMT1 and reduces the direct binding of DNMT1 to the promoter region of the SMAD3 gene, thus inhibiting the activation of signaling by multiple downstream immune signaling pathways (43). In colon cancer, SUV39H1 negatively regulates Fas transcription and impairs the sensitivity of tumor cells to CTL Fas L-mediated cytotoxicity (35). More importantly, SUV39H1 has a non-negligible role in the dysfunction of tumor-infiltrating cells (CTL). It deprives effector T cells of their long-term memory reprogramming capacity (44) and induces SMAD2/3 inhibition of T cells to produce IL-2-mediated immune modulation (45). In conclusion, the inhibition of tumor cell gene expression by SUV39H1 under pathological conditions and its central role in suppressing the killing and memory functions of effector T cells provide new evidence in support of its effectiveness.

#### SETD2

SETD2 is the only human gene responsible for the trimethylation of histone H3 lysine 36 (H3K36me3) that interacts with RNA polymerase II (68, 69). Although there is clear evidence that SETD2 is abnormally expressed in various tumors, its causal relationship with tumorigenesis is still unclear. In the analysis of clinical sample, mutations in SETD2 led to the enrichment of tumor cell surface mutation-specific neoantigens, such as mutational load (TMB) microsatellite instability-high (dMMR/MSI-H). In addition, these patients with SETD2 mutated cancer were accompanied by transcriptional upregulation of genes associated with immune activity (46). Another clinical analysis of lung adenocarcinoma found many SETD2 gene mutations and significantly higher IFN-γ expression in the PD-L1 high-expression group (47). Furthermore, an experimental study in renal cell carcinoma found that SETD2 acts as a transcription factor regulating E3 ubiquitin ligase FBW7 target gene expression, causing altered PD-L1 expression levels and promoting CD4^+^ and CD8^+^ T cell infiltration and enhancing the anti-tumor effects of PD-1 antibodies (48). Based on the above studies, mutations in SETD2 are significantly correlated with tumor immune-specific genes and can drive tumor immunophenotypic alterations. However, extensive experimental studies are still needed to identify specific regulatory mechanisms of SETD2 on immune-related factors, which could provide new insights into the heterogeneous immune treatment of individual tumor patients.

#### KMT2 family

The histone-lysine N-methyltransferase 2 (KMT2) family of proteins is one of the most common mutations in human genome and confers the key functions of chromatin modifiability and DNA accessibility by modifying lysine 4 (H3K4) in the H3 tail of histone H3 (70). The current anti-tumor effects involving the KMT2 family are mainly focused on investigating immune checkpoints. In pancreatic cancer, inhibition of MLL1(KMT2A)activity or silencing expression reduces H3K4me3 levels in the CD274 promoter region and downregulates PD-L1 expression. Moreover, a KMT2A inhibitor combined with anti-PD-L1 or anti-PD-1 antibodies can effectively restrain the growth of a mouse model of pancreatic tumor in a Fas L- and CTL-dependent manner (49). Also, KMT2D is the main mutated gene in PD-L1-positive patients with hepatocellular carcinoma, whose large accumulation may lead to the ineffective response of PD-1 reagents (50). Frequent mutations in KMT2D have also been observed in non-small-cell carcinomas, along with mutations in TP53 (51). The response to immune checkpoint inhibitor (ICI) therapy is mainly influenced by intracellular tumor factors (e.g., tumor mutational load and microsatellite instability) and the tumor microenvironment. In an analysis of the immune assessment of ICI-treated patients through the Biocredit database, KMT2D was identified to have a critical role in a variety of tumor such as bladder cancer (52), esophageal cancer (53), gastric adenocarcinoma (54), lymphoma (56), and head and neck cancer (57, 71). These findings confirm that the KMT2 family is one of the drivers of immune escape. Alterations in its family-related genes may serve as predictive biomarkers for immunotherapy and help us to understand the prognostic effect of immune checkpoint therapy.

#### DOT1L

DOT1L (telomere silencing interference; also known as KMT4), which mainly catalyzes the methylation of H3K79, leads to gene mutations and impairs the interaction between Sir2 and Sir3 in the telomeric region (71). Inhibition of its catalytic activity has been widely used in cancer therapy. Recent studies have suggested that DOT1L is a central player in CD8^+^ T cell physiology, ensuring the activation of normal T cell receptor signaling and related signaling pathways that control CD8^+^ T cell differentiation. In the CD4-CRE transgenic mouse model, deletion of the DOT1L gene inhibited CD8^+^ Tcells apoptosis, as well as TNF and INF-γ expression. Furthermore, inhibition of DOT1L increased the threshold for TCR activation in T cells (58). Another study suggested that the loss of DOT1L directly impairs TCR/CD3 expression, resulting in an impaired immune response (59). Furthermore, DOT1L controls the subset differentiation of Foxp3^+^ regulatory T cells during carcinogenesis, reducing local inflammatory production in the microenvironment (60). The above results suggest that DOT1L is an important epigenetic target for regulating allogeneic T-cell responses, affecting the amount of immune cell infiltration, the direction of cell differentiation, and the secretion of immunomodulatory factors.

### Protein arginine methyltransferase (PRMT) and tumor immunity

**Figure 2:**
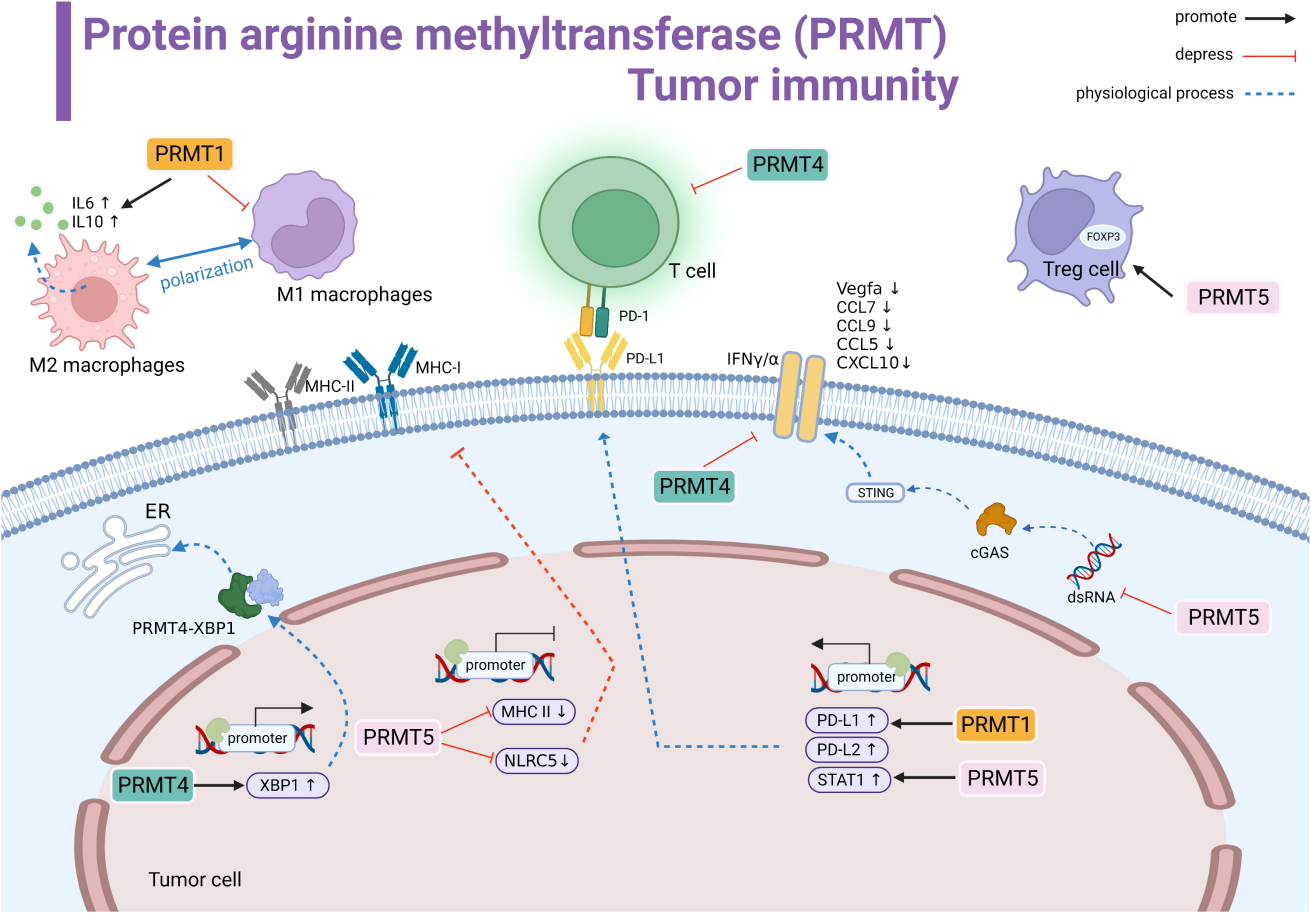
Protein arginine methyltransferase (PRMT) involved in tumor immune summary. PRMT1 regulates M2 macrophage polarization and promotes the transcription of IL-6 and IL-10. PRMT1 promotes the transcriptional level of PD-L1. PRMT4 negatively regulates T cells. PRMT4 promotes the transcription of XBP1 and forms the PRMT4-XBP1 complex to activate the endoplasmic reticulum stress pathway. PRMT5 inhibits the transcription of MHCI and MHCII and suppresses antigen recognition. PRMT5 promotes STAT1 expression to promote PD-L1 expression levels. In the cytoplasm, PRMT5 inhibits the dsRNA-cGAS-STING pathway, downregulates the interferon pathway, and downstream genes Vegfa, CCL7, CCL9, CCL5, and CCL10 expression are suppressed.PRMT4 inhibits IFNγ/α.PRMT5 promotes the immunosuppressive function of Foxp3 regulatory T cells. Black line indicates promotion, red line indicates suppression, and dashed line indicates physiological function.

As a common post-translational modification, PRMT can catalyze the transfer of methyl groups from S-adenosine methionine (AdoMet) to the guanidine nitrogen atom of arginine. It can also affect the methylation status of the cancer genome, leading to activation or inhibitory recruitment of transcriptional mechanisms that are dysregulated in most tumors (72). In recent years, the development of PRMT-targeted drugs has been widely used in cancer therapy. Considering that PRMT1, PRMT4, and PRMT5 have the highest expression in cancer, their immunosuppressive effect have been well investigated (**Table 2**) (**Figure 2**).

**Table 2:** Related functions of arginine methylase and tumor immunity.

Protein	Tumor type	Regulate cell	Target	Mechanistic	References
PRMT1	N/A	CD8^+^ T cell	N/A	Affects the anti-tumor activity of T cells	(73)
	Hepatocellular carcinoma	Tumor cell/Macrophage	PD-L1,PD-L2	Regulates PD-L1 and PD-L2 expression	(74)
	Pancreatic ductal adenocarcinoma	Tumor cell	PD-L1	Promoting the expression of PD-L1	(75)
	Hepatocellular carcinoma	Macrophage	IL-6,IL-10	Control both IL-6 and IL-10 expression and the downstream activation of STAT3, affecting the polarization levels	(76)
PRMT4	Pan-cancer	N/A	N/A	Participate in the regulation of immune response and infiltration	(77)
	Ovarian cancer	Tumor cell	XBP1	Form a complex with XBP1s to regulate their target gene expression, thus determining the ER stress response by controlling the IRE1α/XBP1s pathway	(78)
	Triple-negative breast cancer	Tumor cell	BAF155	Induction of BAF155 methylation and repression of interferon α/γ pathway genes	(79)
	Non-small cell lung cancer	Tumor cell	circHMGB2	As a cicrHMGB2 downstream gene, inhibiting the type 1 interferon response	(80)
	Carcinoma of colon	Tumor cell	N/A	Inhibition to achieve better immune infiltration	(81)
	Pan-cancer	Tumor cell/CD8^+^ T celll	N/A	Shape the immunosuppressive environment	(82)
PRMT5	Melanoma	Tumor cell	NLRC5	Inhibition of the transcription of NLRC5, modulating the genes implicated in MHCI antigen presentation	(83)
	Hepatocellular carcinoma	Tumor cell	CIITA,CD74	Increasing the enrichment of H3R8me2 and H4R3me2 at the CIITA and CD74 promoters, regulates MHC II expression	(84)
	Lung cancer	Tumor cell	CD247	Increases H3R4me2 deposition at the CD274 promoter site and represses gene expression	(85)
	Cervical carcinoma	Tumor cell	STAT1	The expression of both STAT1 and PD-L1 is driven by the IFN/JAK/STAT1 pathway	(86)
	N/A	CD8^+^ T cell	Blimp1	Klrg1 CD8 + Tcell differentiation was inhibited by deposition at the H4R3me2s and H3R8me2s sites of Blimp	(87)
	N/A	CD8^+^ T cell	AKT	Impact on the metabolic reprogramming of cells through the AKT/mTOR signaling pathway	(88)
	N/A	Treg cell	FOXP3	Increase signaling to FOXP3 dimethylation to promote Treg function and migration capacity	(89)

#### PRMT1

Protein arginine methyltransferase 1 (PRMT1) is the main type I PRMT. Many experimental studies have shown that PRMT1 is overexpression or has an shear state in many cancer types (90). Using a genome-wide CRISPR immune screening system to screen for tumor-intrinsic factors that modulate tumor cell sensitivity to T cell-mediated killing, Hou J et al. identified PRMT1 as an intrinsic factor affecting T cell transport and lethality. The possible mechanism is the altered RNA levels of the cytokines/chemokines (73). In some tumor types, PRMT1 is an important regulator of the immune checkpoint pathway. In human hepatocellular carcinoma (HCC), PRMT1 expression is positively correlated with both PD-L1 and PD-L2 immune checkpoint expression (74). Similarly, PT1001B (PRMT1 inhibitor) enhances antitumor immunity by inhibiting PD-L1 expression on tumor cells, upregulating tumor-infiltrating CD8^+^ T lymphocytes. When the anti-PD-L1 monoclonal antibody was combined with PT1001B, the proportion of tumor-infiltrating effector cells was significantly increased in mice, and resistance to anti-PD-L1 treatment was well reversed (75). In addition, PRMT1 can protect the tumor cells, which can induce macrophages to assist in immune escape. Inhibition of PRMT1 in mice led to the inhibition of IL6 signaling and downstream STAT3 activation and decreased the number of tumor cells and M2 type macrophages (76). Taken together, these studies suggested that effective inhibition of PRMT1 can control T cell-mediated tumor killing and can effectively remodel the tumor immune microenvironment.

#### PRMT4

Protein arginine methyltransferase 4 (PRMT4), also known as coactivator-associated arginine methyltransferase 1 (CARM1), has a carcinogenic role in human cancer and is closely involved in the process of tumor growth and immune tolerance (91). CARM1 is overexpressed in different tumors and negatively associated with CD8^+^ T cells. It can also be used as a potent biomarker for pan-cancer prediction (77). In ovarian cancer, CARM1 acts as a transcriptional activator to promote XBP1 target gene expression. CARM1 and interacts with XBP1 to modulatie the ER stress response in the IRE1α/XBP1 pathway, triggering an immunosuppressive environment (78). Furthermore, CARM1 mainly targets BAF155 in triple-negative breast cancer by inhibiting the interferon pathway to inhibit the host immune response (79). Similarly, CARM1 is positively regulated by circHMGB2, which inhibits type I interferon responses and downstream genes. EZM2302 (a CARM1 inhibitor) and anti-PD-1 antibody significantly inhibited the immunosuppressive environment *in vivo* shaped by tumor growth in mice and reduced the efficacy of anti-PD-1 monotherapy in non-small cell lung cancer (80). In a mouse colon cancer model, inhibitors targeting CARM1 were effective in arresting solid tumor progression and enhancing immune infiltration (81). In addition, the inactivation of the CARM1 gene in T cells can increase the number of specific memory-like T cell populations in the microenvironment, allowing the body to maintain a continuous and effective immune attack against tumors. EZM2302 (CARM1) enhances the checkpoint blockade sensitivity of CTLA-4 mAb in a synergistic manner (82). Overall, the inhibition of the activity against CARM1 suppresses tumor progression, promotes T-cell infiltration and sustained immune memory, and may be an effective for immunotherapy of drug-resistant tumors.

#### PRMT5

PRMT5 is the major type II arginine methyltransferase, active in a variety of cellular activities, that achieve tumor-promoting effects through methylation-mediated transcription repression, including inhibition of normal expression of the tumor surface antigen proteins in different tumor types (92). For example, in melanoma, PRMT5 activity inhibits NLRC5 transcription and changes the regulation of the expression of genes involved in the presentation of the major histocompatibility complex class I (MHCI) antigen. Meanwhile, PRMT5 interfere with the dsRNA-cGAS-STING pathway to affect type I interferon responses, promoting immune escape (83). In addition, inhibition of PRMT5 promotes the expression of MHC II (84). Treatment with GSK3326595 (PRMT5 inhibitor) plus anti-PD-1 antibody enhanced the anti-tumor response in the mouse organism (83, 84). Thus, targeting PRMT5 may synergize with immune checkpoint therapy to improve therapeutic efficacy. PD-L1 is a key molecule highly expressed in tumor cells that interacts with immune cells to constitute an immunosuppressive environment. In lung cancer, GSK591 drug inhibits PRMT5-induced PD-L1 expression, which then trigger immune resistance (85). Thus, the combination with PD-1 treatment and inhibition and elimination of PRMT5 may promote synergistic inhibition. In contrast, in cervical cancer, PRMT5 promotes cancer progression by increasing the expression of histone H3R2 symmetric dimethylation (H3R2me2s), which is enriched in the promoter region of STAT1 to enhance transcription and drive up-regulation of PD-L1 expression (86).

Furthermore, PRMT5 also acts directly on the host immune cells to maintain cellular physiology and homeostasis, especially on the effector CD8^+^ T cells. PRMT5 can affect the deposition of H4R3me2s and H3R8me2s at the Blimp1 locus and force the differentiation of transient effector CD8^+^ T cells, resulting in a substantial loss of CD8^+^ T cell numbers and function (87). Inhibition of PRMT5 is a “double-edged sword”, its inhibition causes reduced AKT/mTOR signaling, which impairs glycolysis and increases fatty acid utilization after human CD8^+^ Tcells’stimulation leading to metabolic reprogramming (88). In addition, PRMT5 can interact with the FOXP3 transcription factor in Tregs to maintain the functional stabilization of Treg cells (89). In conclusion, given the selective role of PRMT5 in the tumor microenvironment, more attention should be paid to the mechanism of side effects in immune cells, and combined immunotherapy may maximize the efficacy.

### Classification and biological functions of histone demethylases(HDMs)

With the progress of science and technology, almost all histone lysine methylation sites have been found to be reversible. To date, two classes of histone demethylases have been identified, mainly the lysine-specific demethylase-1 (LSD1) family and the jumonji (JmjC) domain-containing family (93). LSD1, which was identified first acts only on monomethylated and dimethylated lysines (94). The JmjC family is another class of JmjC domain-containing Fe (II). Ketoglutarate-dependent enzymes are divided into different species according to the sequence homology of the JmjC domain and the overall structure of the related motifs. Thus far, those active against H3K4, H3K9, H3K27, H3K36, and H4K20 have been identified (95). Their special structure allows them to function together with many other biological macromolecules (96).

**Figure 3:**
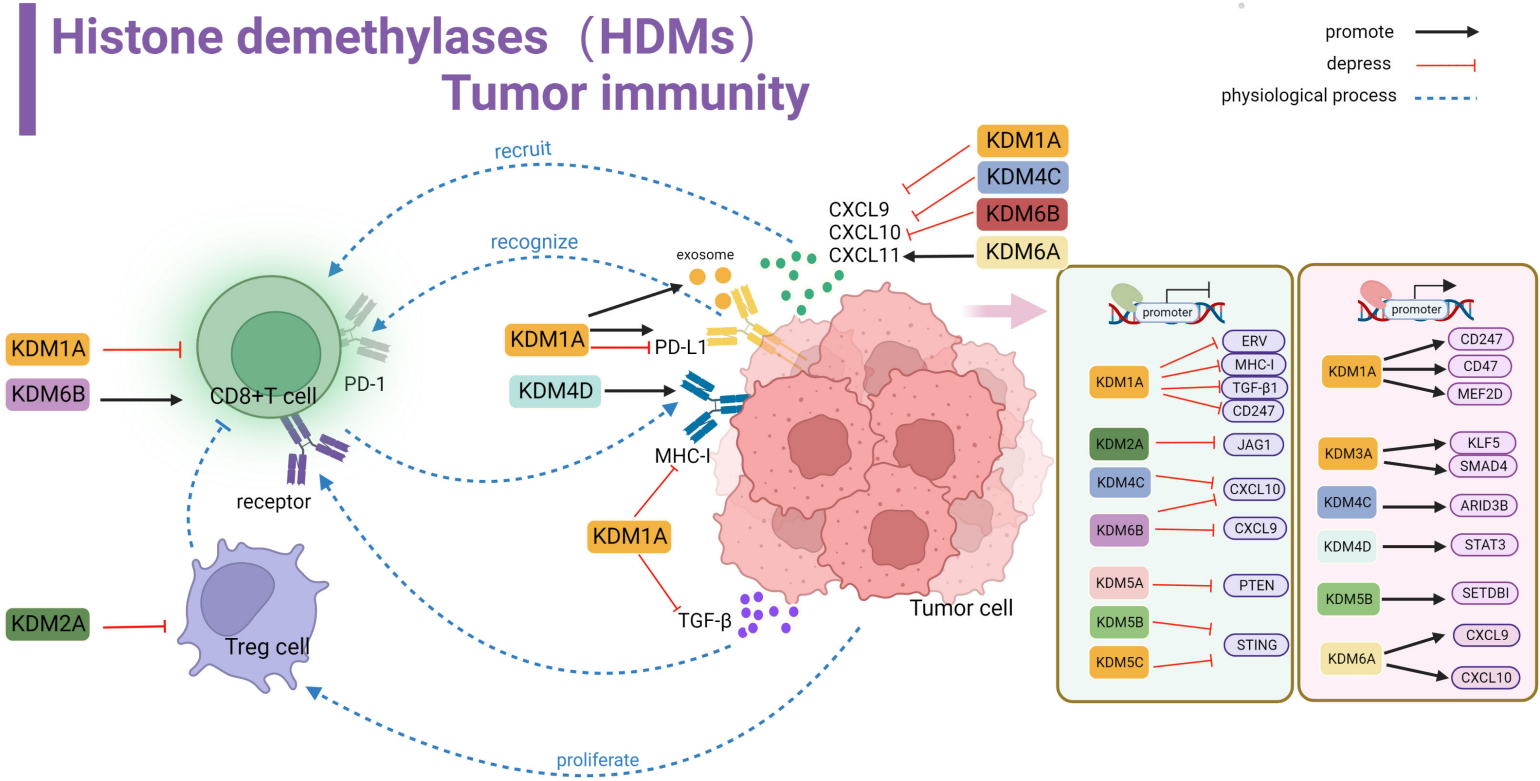
Histone demethylase(HDMs) involved in tumor immune summary. In tumor cells, KDM1A, KDM2A, KDM4C, KDM5A, KDM5B, KDM5C and KDM6B negatively regulate key genes and signaling pathways involved in stimulating T-cell anti-tumor immunity, including ERV, MHC-I, TGF-β, CD247, JAG1, CXCL10,9,STING and PTEN, affecting cellular KDM1A, KDM3A, KDM4C, KDM4D, KDM5B, KDM5A positively regulate related proteins involved in activating tumor surface antigens, including CD247,CD47 and other surface antigens, or by promoting MEF2D, KLFS, SMAD4, STAT3, ARID3B, SETDBI to promote or activate downstream KDM5A promotes CXCL9 and CXCL10 recruitment of T cells into the microenvironment. kdm1A inhibits TGF-β binding to T cell-associated receptors and suppresses MHC-I antigen expression. KDM1A promotes PD-L1 expression in exosomes. kdm1A and KDM6B affect T cell function. kdm2A alters the activity of regulatory T cells.

Histone demethylases do not change the DNA sequence, and dynamically regulate in specific chromatin regions. They are important regulators of the physiological functions of embryonic development, gene regulation, cell reprogramming and other physiological functions, and they maintain genome integrity and epigenetic stability (97). Their role in cancer is particularly important, and it is closely related to the pathogenesis of the disease, including the demethylation of the oncogenes/tumor suppressor genes for mastering the cell fate, the enrichment of transcription factors, gene copy number alterations, and increased mutations. Targeting partial demethylases opens up an emerging field for anticancer therapy. In this process, some enzymes also have a prominent role in regulating the immune microenvironment (**Table 3**) (**Figure 3**).

**Table 3:** Related functions of lysine demethylase and tumor immunity.

Protein	Tumor type	Regulate cell	Target	Mechanistic	References
KDM1A	Pan-cancer	Tumor cell	ERV	Suppressing ERV expression and curbing activation, such as dsRNA stress and type I interferon	(98)
	Melanoma/Breast cancer/Small-cell lung cancer	Tumor cell	MHC-I	Inhibition of MHC-I gene expression and reduced antigen presentation	(98–100)
	Cervical cancer	Tumor cell	CD274, CD47	Mediated demethylation of H3K4 in the CD274/CD47 promoter region	(101)
	Hepatocarcinoma	Tumor cell	MEF2D	Promote PD-L1 expression by MEF2D demethylation	(102)
	Gastric cancer	Tumor cell	PD-L1	Altering PD-L1 expression in exosomes did not affect membrane PD-L1 levels	(103)
	Squamous cell carcinoma of the head and neck	Tumor cell	PD-L1	Inhibition of PD-L1 expression	(104)
	Breast cancer	Tumor cell	TGF-β1	Binding to the TGF-1 promoter region, which upregulates its expression	(105)
	Pan-cancer	CD8^+^ T cell	TCF1	The LSD1/CoREST complex physically interacts with TCF1 and antagonizes its transcriptional activity	(106)
	Melanoma/Breast cancer	CD8^+^ T cell	EOMES	Affect the posttranslational level status of the EOMES	(107)
KDM2A	Glioma	Tumor cell	JAG1	Promotes JAG1 demethylation and mediates the proliferation and activity of regulatory T cells	(108)
KDM3A	Pancreatic cancer	Tumor cell	KLF5, SMAD4	In coordination with KLF5, SMAD4 regulates transcription in tumor cells to inhibit anti-tumor immunity	(109)
KDM4A	Squamous cell carcinoma of the head and neck	Tumor cell	N/A	Inhibition of immune-related signaling pathways	(110)
KDM4B	Carcinoma of endometrium	N/A	N/A	Associated with immune cell infiltration and immune checkpoint molecular expression	(111)
	Colon cancer	Tumor cell	HOXC4	PD-L1 expression was induced by the H3K27me3/HOXC4 axis	(112)
KDM4C	Lung cancer	Tumor cell	CXCL10	Promoting the accumulation of H3K36me3 in the CXCL10 promoter region to repress the transcription level of genes affects T cell recruitment	(113)
	Colorectal cancer	Tumor cell	ARID3B	Recruited by ARID3B to activate downstream Notch and PD-L1 expression	(114)
KDM4D	Colorectal cancer	Tumor cell	IFNGR1	Co-activating SP-1 promotes IFNGR1 expression, thereby enhancing STAT3-IRF1 signaling and promoting PD-L1 expression	(115)
KDM5A	Melanoma/Colon cancer	Tumor cell	PTEN	Inhibition of PTEN expression and induction of PI3K-AKT-S6K signaling pathway to increase the PD-L1 abundance in the tumor cells	(116)
KDM5B	Melanoma	Tumor cell	SETDB1	Recruiting the H3K9 methyltransferase SETDB1 to exert antitumor effects	(40)
KDM5B/C	Breast cancer	Tumor cell	STING	Binds to the STING promoter to directly suppress transcription, causing disruption of the cGAS/STING pathway signaling	(120)
KDM6A	Hepatocarcinoma	N/A	N/A	Correlation with the immune infiltration	(123)
	Bladder cancer	N/A	N/A	Negative correlation with immune-related pathways	(124–126)
	Medulloblastoma	Tumor cell	CXCL9,CXCL10	Activates Th-1 type chemokine expression, and enhances T cell recruitment	(127)
KDM6B	Colon cancer	Tumor cell	CXCL9,CXCL10	Inhibition of the expression of both CXCL9 and CXCL10	(26)
	N/A	CD8^+^T cell	GZMB, FasL	Promote the expression of GZMB and FasL effector genes through demethylation	(128)
	N/A	CD8^+^T cell	N/A	Promote cytotoxicity-related gene expression	(129)
	Pan-cancer	N/A	N/A	Associated with TMB, MSI and immune cell infiltration	(130)

#### LSD1 and tumor immunity

Lysine-specific demethylase 1 (LSD1), also known as KDM1A, acts as an H3K4/9me eraser that binds to CoREST or nucleosome remodeling to repress gene transcription (131). LSD1 is highly expressed in most solid tumors, altering tumor immunogenicity and immune response by inhibiting or activating different signaling pathways. Shi et al. first discovered that inhibiting LSD1 can enhance endogenous transcription (EVR) expression, activate dsRNA stress and type I interferon activation, and improve the immunotherapy response of poorly immunogenic tumors (98). More importantly, LSD1 is inversely associated with CD8^+^ T cells in various tumors. In tumor cells, LSD1 largely affects the normal expression of MHC-I protein antigen by inhibiting the MHC-I encoding genes H2-D1 and H2-K2, which leads to the possibility that CD8^+^ T cells do not effectively recognize MHC-I prompting immune escape. The above mechanism has been observed in melanoma, breast cancer, and small-cell lung cancer (98–100)

Conclusions regarding the regulation of PD-L1 expression are inconsistent. In cervical cancer, LSD1 seems to be positively correlated with PD-L1 levels, in which H3K4me2 demethylation directly promoted the increase in PD-L1 expression (101). On the other hand, the demethylation of MEF2D in HCC indirectly promotes the PD-L1 expression, and this process is competitively inhibited by has-miR-329-3p (102). Moreover, in gastric cancer, LSD1 increases the level of PD-L1 found in exosomes and is transported to T-cell expression to inhibit tumor immunity (103). In contrast, LSD1 significantly suppresses the PD-L1 expression level in HNSCC (104). The surprising finding is that using the LSD1 inhibitor alone, despite its effective tumor suppression, the resulting exogenous TGF-1 binding to the CD8^+^ T cell surface receptors inhibits the cytotoxic effects (105), which may be one of the reasons why the clinical effects of LSD1 inhibitors are suboptimal. Alternatively, LSD1 performs an epigenetic program within CD8+ T cells. On the one hand, it inhibits the transcription of the progenitor phenotype gene TCF1, disrupting the progenitor cell population (106). On the other hand, eomesodermin (EOMES), a transcription factor associated with the regulation of T cell failure, promotes T cell dysfunction (107). These make T cell depletion fast and unsustained recovery, resulting in poor persistence of PD-1 blocking therapy. Current experimental data suggest that treatment with LSD1 inhibitors (ORY-1001, SP2509 or GSK2879552) in combination with PD-1/PD-L1 monoclonal antibodies enhances *in vivo* immunogenicity and has a long-term response (101, 104, 106).

#### JmjC family and tumor immunity

##### KDM2

KDM2 is mainly responsible for the demethylation of the H3 lysine 36(H3K36) residues, and its family members include KDM2A and KDM2B (132). In glioma, LncRNA HOXA-AS2 promotes KDM2A expression by binding to miR-302a, thus recruiting H3K4me3 to demethylate JAG1 and promoting the proliferation and immune tolerance of regulatory T cells (108). In addition, KDM2A may promote immune body suppression Fumarate as an important metabolite may antagonize inhibitory histones and promote immune regulation (133, 134). In conclusion, KDM2 serves as a considerable therapeutic target.

##### KDM3

KDM3 is mainly composed of KDM3A, KDM3B, and KDM3C, which can specifically catalyze the demethylation of histone H3K9me1/2 (135). Using CRISPR screening in a mouse model of pancreatic cancer, KDM3A was found to be an epigenetic modulator of the response to immunotherapy. KDM3A mainly affects the KLF5 and SMAD4 transcription factor activity, regulates the epidermal growth factor receptor (EFFR) expression, and affects the T cell infiltration and the infiltration of dendritic cell DC (109). This suggests that KDM3A is closely related to the composition of the immune microenvironment. Therefore, eliminating KDM3A could help overcome immunotherapy resistance and enhance sensitivity to therapeutic effects, thereby creating a microenvironment for T-cell inflammation.

##### KDM4

The KDM4 protein family is composed of (KDM4A-C) and KDM4D, and several studies have found them to be overexpressed in cancer and to have the ability to malignant tumor growth (136). Notably, while maintaining tumor growth, they simultaneously suppress the activity of some pathways to interfere with normal immunosuppression. In HNSCC, the knockdown of KDM4A led to the activation of both types I IFN interferon signaling and DNA replication stress signal cGAS-STING, along with the significant upregulation of CXCL9, CXCL10, and CXCL11, and significantly increases the effect of the combined PD-1 blocking treatment (110). KDM4B is also recommended as a clinical prognostic marker and is closely associated with immune cell infiltration and immune checkpoint molecular expression (111). In colon cancer cell culture, KDM4B elevates HOXC4 expression by driving H3K27me3 demethylation to induce the expression of PD-L1, and exogenous miR-15a was able to prevent tumor escape events from occurring (112). Moreover, KDM4C is negatively associated with CD8^+^ T cells in lung cancer; transcription sequencing found that KDM4C mainly downregulates the transcript level of CXCL10 and inhibits T cell recruitment to tumors and killing (113). KDM4C is also involved in the regulation of PD-L1 expression, and the main mechanism is the transcriptional activation of the Notch gene and PD-L1 through ARID3B recruitment to regulate chromatin structure, whereas KDM4D promotes PD-L1 expression through the SP-1/STAT3/IRF1 signaling pathway, assisting the immune escape of in colorectal cancer (114, 115).

##### KDM5

The KDM5 protein family, including KDM5A-C and KDM5D, is responsible for removing histone H3 lysine 4 dimethylation and trimethylation (H3K4me2 and H3K4me3) (116). It is an attractive target in cancer therapy. Several prospective raw letter analyses have shown that KDM5 is closely associated with regulaing immune infiltration and expressing immune-related molecules, and is considered a prospective candidate for epigenetic anti-tumor therapy (117–119). In clinical treatment, some patients have low tumor cell PD-L1 abundance, so they cannot respond well to ICB. One study showed that increased KDM5A gene expression or protein abundance, promoting PD-L1 upregulation to accommodate the PD-1 treatment response, is a valuable clinical response tag (137). In melanoma, high expression of KDM5B can recruit the H3K9 methyltransferase SETDB1 to interact in the suppression of endogenous retrotransposable elements and block subsequent RNA and DNA sensing pathways as well as type I interferon responses, resulting in the inability of the organism to respond positively to tumor rejection and immune responses (40). A similar mechanism has been found in breast cancer. The STING promoter is directly transcriptionally repressed by KDM5B and KDM5C, disrupting the cGAS/STING pathway signaling and failing to activate a robust interferon response (120). Using KDM5 inhibitors reversed the normal transmission of this signaling pathway. It has also been suggested that combining of immunotherapy and KDM5 inhibitors could maximize the anti-tumor immune response, thus representing a potential therapeutic modality of interest.

##### KDM6

The KDM6 subfamily consists of three distinct members, i.e., KDM6A (also called UTX), KDM6B (also called JMJD3), and KDM6C (also called UTY), capable of removing di-and trimethylated H3K27, thereby activating or repressing target gene transcription (121). Its Function is highly dependent on the specific of the cell type pathological environment (122). The molecular basis of KDM6 in tumors is still in its infancy, and only a few studies have addressed this issue. Yet, several studies have shown a high correlation between its mutations and tumor immunity. A functional screen for lysine demethylase in HCC showed that KDM6A is closely associated with immune infiltration (123). In bladder cancer and its subtypes, KDM6A is a more frequently mutated gene, that negatively regulates the signaling pathways of the immune system and suppresses tumor immunity (124–126). In medulloblastoma, KDM6A activates the expression of Th1-type chemokines and promotes cell migration (127). Moreover, KDM6B inhibit CXCL9 and CXCL10 expression in colon cancer and exerts an anti-tumor immune effects (26). In contrast, the effect of KDM6B is positively regulated for CD8^+^ T cells. KDM6B can promote the differentiation of mature CD8^+^ T cells by demethylating the expression of GZMB and FasL (128). Inhibition of KDM6B resulted in reduced of toxicity-related genes in CD8^+^ T cells (129). Little experimental support exists for the specific mechanism of KDM6B in tumor progression and immune cell infiltration. However, available pan-cancer analyses suggest that KDM6B expression is associated with TMB, MSI and immune cell infiltration, and influences the response to immunotherapy and clinical outcome (130).

### Conclusions and outlook

In the past decade, human cancer prevention and treatment have entered a new era with the emergence of immunotherapy. In the process of gradually understanding the potential mechanism of tumor cell occurrence and development, to the mechanism of killing malignant cells and avoiding the effect of the immune system, researchers have also developed corresponding therapeutic drugs for clinical practice, including immune checkpoint inhibitors, epigenetic targeted drugs, etc. Nevertheless, the low response rate and immune resistance in practical clinical applications led to identification of so-called “cold tumor”.

The concentrated research on histone methylation modifying enzymes in epigenetics advances our new understanding of “cold tumors” in human cancer, and builds the bridge between tumor cells and immune cells, promoting a deeper understanding of the complexity and diversity of the tumor immune microenvironment. Current studies on the involvement of histone methylase and demethylase in anti-tumor immunity mainly includes (1): regulation of tumor immunogenic antigen expression; (2) their influence on the activation of immune-related pathways; (3) regulation of expression of chemokines/cytokines and induced immune-related factors; (4) regulation of immune cells, including immune cell activation, immune cell depletion and functional remodeling, and immune memory. The above regulatory mechanisms provide a more comprehensive picture of the facilitative/suppressive immune microenvironment shaped by aberrant histone methylation modifications at the transcriptional and translational levels. Furthermore, the contribution of histone methylation modifications for tumor immune escape mechanism, immunotherapy tolerance mechanism, and immune stress has brought new perspectives and approaches for solving the “cold tumor” dilemma.

The above studies are still in their infancy but provide a solid theoretical basis for future preclinical and clinical development of combination therapies using epigenetic modulators and immunotherapeutic agents and show great potential. This will be a new therapeutic paradigm targeting improved and enhanced immune efficacy. We expect that based on the rapid development of immunogenomics, immunoproteomics, and immunobioinformatics, the complex structures in the tumor immune microenvironment will be revealed more comprehensively in the future. Together with the development of research on immune features in preclinical tumor models, this will greatly improve our understanding of the role of histone methylation in the immune microenvironment, facilitating clinical translation and the construction of precise therapeutic systems. Therefore, the development of this field is an important breakthrough to improve the efficacy of immunotherapy for the benefit of more patients. Based on the current research, we still need further studies to explore the role of histone methylation mutations in the regulation of immune resistance in different types of tumors. Meanwhile, the combination of single cell sequencing and spatial transcriptome sequencing will fully reveal the importance of histone methyl esterases in the tumor microenvironment, providing finer evidence to support the mechanism of epigenetic involvement in immune regulation. In addition, experimental models of combining multiple histone methylation modulators with immunotherapeutic agents will be developed, and rational and less toxic optimization protocols will be sought to advance clinical practice.

In conclusion, understanding the regulatory mechanisms of histone methylation modifying enzymes will improve immunotherapy.

## Author contributions

YZ and JC collected relevant literature, prepared data, and drafted the manuscript. HL and RM participated in the design of this review. RH, FF and XX participated in the summary and drawing of tables and pictures. XL and JD made strict revisions to the manuscript. All authors read and approve the final draft.
